# (Multi-) omics studies of ILC2s in inflammation and metabolic diseases

**DOI:** 10.3389/fcell.2024.1473616

**Published:** 2024-10-28

**Authors:** Maria Kral, Emiel P. C. van der Vorst, Christian Weber, Yvonne Döring

**Affiliations:** ^1^ Institute for Cardiovascular Prevention (IPEK), Ludwig-Maximilians University Munich, Munich, Germany; ^2^ DZHK (German Center for Cardiovascular Research), Partner Site Munich Heart Alliance, Munich, Germany; ^3^ Aachen-Maastricht Institute for CardioRenal Disease (AMICARE), Interdisciplinary Center for Clinical Research (IZKF), Institute for Molecular Cardiovascular Research (IMCAR), RWTH Aachen University, Aachen, Germany; ^4^ Department of Biochemistry, Cardiovascular Research Institute Maastricht (CARIM), Maastricht University Medical Centre, Maastricht, Netherlands; ^5^ Munich Cluster for Systems Neurology (SyNergy), Munich, Germany; ^6^ Department of Angiology, Swiss Cardiovascular Center, Inselspital, Bern University Hospital, University of Bern, Bern, Switzerland; ^7^ Department for BioMedical Research (DBMR), University Hospital, University of Bern, Bern, Switzerland

**Keywords:** ILC2, metabolism, metabolic diseases, omics, single-cell RNA sequencing

## Abstract

Type 2 innate lymphoid cells (ILC2s) have emerged as pivotal regulators in the pathogenesis of diseases, with their roles in inflammation, metabolism, and tissue homeostasis becoming increasingly recognized. This review provides an overview of the current understanding of ILC2s in inflammation and metabolic disorders, including their functional contributions. Moreover, we will discuss how these cells adapt their metabolic processes to support their function and survival and how their metabolic requirements change under different physiological and pathological conditions. Lastly, we will review recent omics studies that have provided insights into the molecular and cellular characteristics of ILC2s. This includes transcriptomic, proteomic, and metabolomic analyses that have elucidated the gene expression profiles, protein interactions, and metabolic networks, respectively, associated with ILC2s. These studies have advanced our understanding of the functional diversity of ILC2s and their involvement in metabolic disease.

## Introduction

Innate lymphoid cells (ILCs) belong to a newly discovered, rare cell population of the innate immune system. In the past years, it has become clear that they exert important functions in host defense against invading pathogens, in guiding immune reactions and in tissue immune homeostasis (Kim et al., 2021; Spits et al., 2013; Walker et al., 2013). Since their discovery in 2010, several subsets have been characterized based on their phenotype and functional properties (Artis and Spits, 2015; Spits and Cupedo, 2012). ILCs develop from a common lymphoid progenitor (CLP) that gives rise to group 1 ILCs (ILC1s), ILC2s, and ILC3s. Due to the similarities of transcription factor and lineage-specific cytokine expression to T helper cells, they are considered to be their innate counterparts (Spits et al., 2013). For example, T-bet and interferon (IFN)-γ are expressed by both ILC1s and Th1 cells. While for Th2 cells, shared GATA binding protein 3 (GATA3) expression and type-2 cytokine production (Interleukin (IL)-4, IL-5, and IL-13) makes ILC2s their innate counterparts, ILC3s require RAR-related orphan receptor gamma t (RORγt) for their development and are able to produce Th17- and Th22-like cytokines and are therefore thought to be the innate equivalent of Th17 and Th22 cells.

Among ILCs, ILC2s are important mediators of tissue remodeling and immune homeostasis by orchestrating immune crosstalk between cells of the innate and adaptive immune system (reviewed in (Kral et al., 2023)). These cells mainly reside at mucosal barriers or within tissues, and therefore serve as first line of defense for invading pathogens. Here, release of the key activating epithelial cytokines IL-25, IL-33, and thymic stromal lymphopoietin (TSLP) leads to the activation of ILC2s. However, the expression of their receptors (IL-25R, ST2, and TSLPR, respectively) varies across different tissues and other activating cytokines have been identified depending on the localization (Ricardo-Gonzalez et al., 2018). For instance, it has been shown that skin ILC2s express low levels of these receptors and are rather activated by IL-18 (Ricardo-Gonzalez et al., 2018). Importantly, ILC2 are also located in metabolically active tissues, such as the adipose tissue, where they are essential in maintaining immune homeostasis in the tissue (Moro et al., 2010; Sasaki et al., 2019). This is ensured by the secretion of IL-5 and IL-13, resulting in the recruitment of anti-inflammatory M2 macrophages and eosinophils.

Dysfunction of ILC2s has been implicated in the development of various diseases. Due to their local availability in metabolic active tissues, several studies pointed towards a role of impaired ILC2 activity in the pathogenesis of metabolic diseases (Brestoff et al., 2015; Michailidou et al., 2022; Molofsky et al., 2013). Such diseases are characterized by disrupted energy metabolism and include Type 2 Diabetes (T2D), hyperthyroidism and hypothyroidism, and the metabolic syndrome, among others. Importantly, patients suffering from the metabolic syndrome have an increased risk of developing atherosclerotic cardiovascular disease (ACVD) (Li et al., 2021). ACVD is characterized by plaque buildup in the artery walls due to accumulation of lipids and immune cells, resulting in enhanced secretion of pro-inflammatory mediators at the site of inflammation (Bullo et al., 2003). As a result, thickening of these vessels can, for example, cause coronary heart disease, cerebrovascular disease, depending on the location. Lifestyle modifications can reduce the risk for this chronic inflammatory condition; however, CVDs are still one of the leading causes of death. Current medical interventions only treat the symptoms but not the origin of the disease. Therefore, a better understanding of the underlying mechanisms leading to this condition could critically contribute to the development of more specific, beneficial preventive and therapeutic approaches.

In recent years, the perivascular adipose tissue (PVAT) surrounding the artery vessel walls, has been recognized as important regulator of vascular biology. In a healthy state, PVAT regulates vascular tone and intravascular thermoregulation (Stanek et al., 2021). However, sustained inflammatory conditions contribute to an inflammatory milieu by the release of pro-inflammatory mediators (e.g., TNF, IL-6) (Stanek et al., 2021; Guzik et al., 2006; Guzik et al., 2007). Moreover, reactive oxygen species (ROS) production in response to increased oxidative stress in PVAT leads to damage of endothelial cells (Barp et al., 2021). In addition, dysregulation of vascular tone under these conditions can cause an increase in blood pressure (Ma et al., 2023). Collectively, PVAT dysfunction has been shown to be linked to cardiovascular and metabolic disorders. Notably, ILC2s are highly abundant in PVAT and due to their anti-inflammatory properties, they might impact vascular health (Newland et al., 2017). Accordingly, increased release of pro-inflammatory cytokines in dysfunctional PVAT critically modulates ILC2 functions by reducing their ability to produce IL-5 and IL-13. Therefore, the interaction of ILC2s and PVAT is an important factor in the development of chronic vascular inflammation leading to CVDs. However, this research area is still emerging, and more studies are necessary to fully understand the mechanisms of ILC2-PVAT interactions in the pathogenesis of CVDs.

Hence, dysregulated functions of ILC2s in regulating metabolic homeostasis in adipose tissue (AT) can critically contribute to obesity and related metabolic disorders. Therefore, the next section will focus on ILC2 metabolism and how its dysregulation impacts inflammation and the progression of diseases.

## ILC2 metabolism

In this regard, the field of “immunometabolism” has gained a lot of attention in the past years. Importantly, immune cells have an increased demand of energy supply for their functionality, differentiation, and survival and lack of this supply might impact the development of diseases (Aderinto et al., 2023). It is thus important to gain a better understanding of immune cell metabolism in order to implement these findings for novel therapeutic strategies of immune response modulation.

**FIGURE 1:**
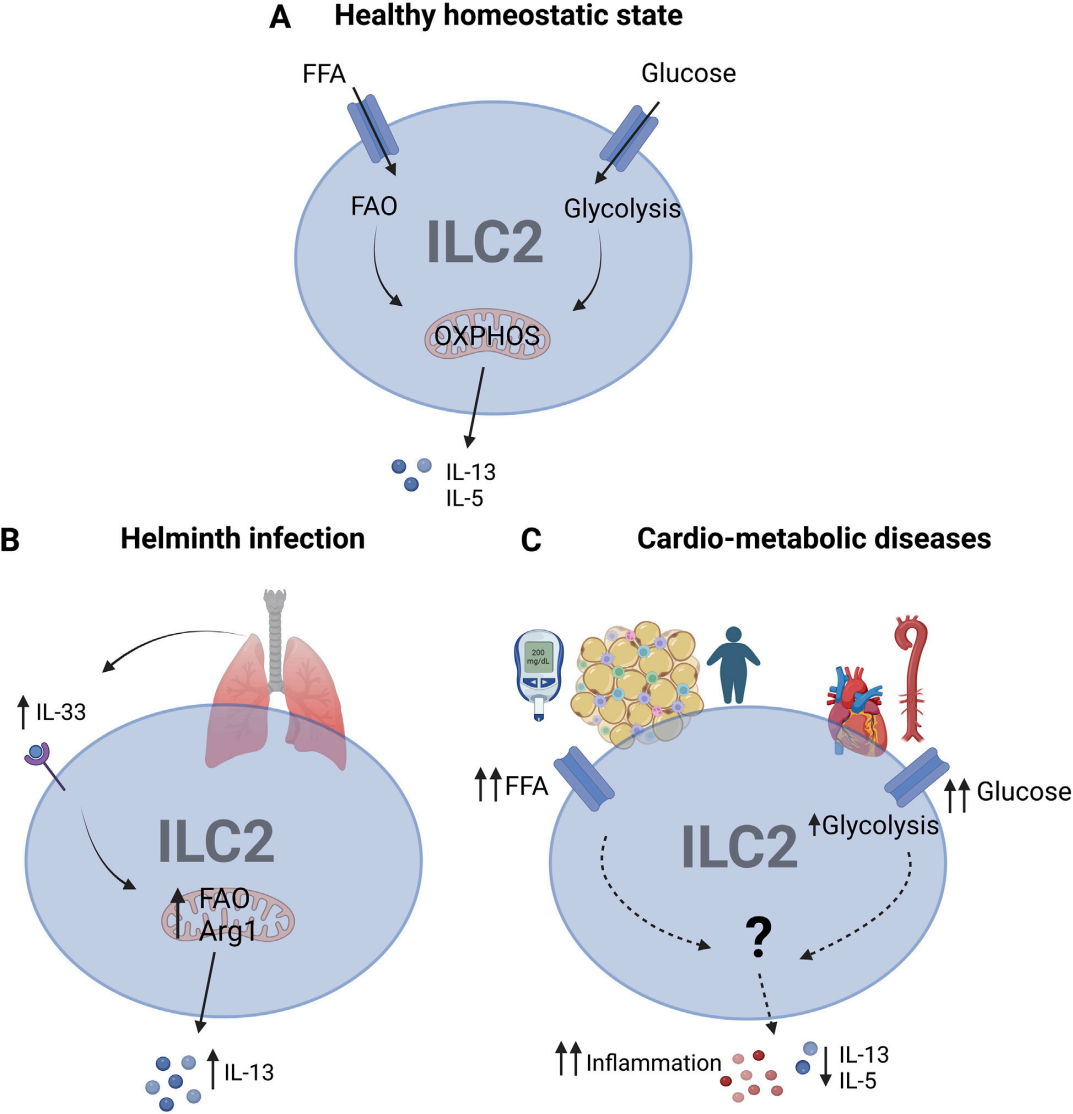
ILC2 metabolism in healthy and diseased state **(A)** In healthy homeostatic state, ILC2s use glycolysis and FAO to fuel OXPHOS for the production of IL-5, IL-13 cytokines to maintain their survival. **(B)** Upon helminth infection in the lung, IL-33 is released which in turn activates ILC2s, leading to FAO and Arg1 activity for IL-13 production in order to enhance the anti-inflammatory and tissue-repair functions of ILC2s. **(C)** In metabolic disorders, impaired metabolism due to high glucose and free fatty acids (FFA), ILC2 metabolism is disrupted, partly leading to increased glycolysis to manage the excess of glucose Abbreviations: Arginase-1 (Arg1), Fatty acid oxidation (FAO), Free fatty acids (FFA), Innate lymphoid cells type 2 (ILC2s), Interleukin (IL), oxidative phosphorylation (OXPHOS). Made with biorender.com. The “?” indicates that there is a yet unknown mechanism.

In order to be functional, ILC2s rely on a high metabolic state to fuel their activity. For most immune cells glucose is the primary source for their activation and effector functions in producing cytokines (Soto-Heredero et al., 2020). Intriguingly, depending on the activation state and the environmental milieu, ILC2s can also use mitochondrial oxidative phosphorylation (OXPHOS) to meet their metabolic demands (Surace et al., 2021). Alternatively, ILC2s can also depend on fatty acid oxidation (FAO) in order to provide a sustained source of ATP required for their survival (Wilhelm et al., 2016) (Figure 1A).

Even though our understanding of immune metabolism of ILC2s is still in its infancy, several studies have already revealed key metabolic pathways for their activity. First studies showed that upon helminth infection ILC2s increase their demand on FAO in order to produce IL-13 (Wilhelm et al., 2016). Conversely, under steady state conditions ILC2s do not rely on FA metabolism for maintaining their numbers (Wilhelm et al., 2016). Moreover, it could be demonstrated that ILC2s use arginine and branched-chain amino acids to fuel their energetic demands upon proliferation and activation (Monticelli et al., 2016). In this regard, a study found evidence that the enzyme arginase-1 (Arg1) regulates ILC2 metabolism during acute and chronic lung inflammation (Karagiannis et al., 2020) (Figure 1B). More specifically, the amino acid transporter SLC7A8 has been identified as important regulator of key metabolic pathways in ILC2s and that its deficiency in lymphocytes reduced type 2 immune responses to helminth infection (Panda et al., 2022; Hodge et al., 2023).

These different energy sources are used depending on the local fluctuations of metabolic nutrients and thus, ILC2s have to adapt to such environmental changes. Moreover, ILC2s reside in various tissues such as skin, lung, and AT which have tissue-specific nutrients available (Yu et al., 2022). This flexibility and adaptive capacity upon metabolic changes of ILC2s makes them an attractive candidate for the development of therapeutic interventions. Previous studies could highlight how ILC2s are able to respond to the availability of different nutrients in a tissue and disease-specific context (Yu et al., 2022). Accordingly, their metabolic state has been implicated in different metabolic diseases as detailed below.

## ILC2 metabolism in disease

### - Cardio-metabolic disorders

Metabolic disorders are a result of disrupted energy metabolism, leading to diseases such as obesity and T2D. Diet-induced obesity is a complex condition resulting from fat accumulation and its prevalence is rising dramatically, making it a growing epidemic (Mitchell et al., 2011). Chronic low-grade inflammation that occurs in obesity affects multiple organs in the body, such as the AT, pancreas and the liver (Park et al., 2014). Since ILC2s are present in those tissues in order to maintain immune homeostasis, they have been described to play a role in these metabolic disorders. In 2015, ILC2s have been identified in human white adipose tissue (WAT), where they are involved in maintaining metabolic homeostasis (Odegaard and Chawla, 2015). Importantly, Brestoff *et al.* could link decreased ILC2 responses in WAT with obesity in both mice and men (Brestoff et al., 2015). Specifically, IL-33 induces the recruitment of uncoupling protein 1 (UCP1) positive beige adipocytes in WAT. This process is referred to as “beiging” or “browning” to regulate caloric expenditure (Cohen et al., 2014; Harms and Seale, 2013; Shabalina et al., 2013) and IL-33-induced beiging was dependent on ILC2s (Brestoff et al., 2015). Similarly, one study found that the lack of ST2, the receptor for IL-33, led to a decrease of ILC2s in WAT (Okamura et al., 2021). Intriguingly, ST2 deficiency on ILC2s increased the numbers of ex-ILC2s, resembling ILC1-like features, which is associated with decreased beiging in WAT and concomitant impairments in energy metabolism (Okamura et al., 2021). These studies highlight the importance of ILC2s in controlling metabolic homeostasis in AT.

Furthermore, chronic obesity can cause the development of several obesity-associated diseases. T2D develops as a result of persistently increased blood glucose levels, eventually causing damage to other organs (Galicia-Garcia et al., 2020). Notably, hyperglycemia in patients with T2D can also cause metabolic stress on ILC2s, consequently altering their cellular metabolism (Painter and Akbari, 2021) (Figure 1C). In this regard, one group found that liver ILC2s are involved in regulating glucose homeostasis (Fujimoto et al., 2022). However, studies in the context of CVDs still need to be published. Similarly, alterations in lipid metabolism in T2D, e.g., enhanced levels of free fatty acids (FFA) can modulate ILC2s (Painter and Akbari, 2021) (Figure 1C). Here, the energy sensor mammalian target of rapamycin (mTOR) controls the enzymes peroxisome proliferator activated receptor gamma (PPARγ) and diacylglycerol-O-acyltransferase 1 (DGAT1) in storing FFAs in droplets within the cells (Karagiannis et al., 2020). Consequently, impaired lipid processing leads to accumulation of FFA in adipose ILC2s, resulting in lipotoxicity.

Similar impairments in ILC2 metabolism can be observed in the context of CVDs (Roberts et al., 2022). Importantly, impaired lipid metabolism drives a metabolic shift to increased glucose intake as a compensatory mechanism (Figure 1C). Current understanding suggests that this immunometabolic shift might impact the functionality and survival of ILC2s, thereby exacerbating tissue inflammation in CVDs (Yu et al., 2022). Therefore, tight control of glucose and fatty acid metabolism is essential for ILC2 function and survival. Even though impairments in ILC2s have been linked to CVDs, the mechanisms of their dysfunction remain poorly understood.

Targeting metabolic pathways of different cell types as novel therapeutic approach offers possibilities to fine-tune treatment efficacy and can help to restore normal metabolic functions. Therefore, such therapies hold significant promise for a variety of diseases. Since there are significant differences in energy production pathways in ILC2s depending on the tissue and disease context, a better understanding on how they fuel their metabolic needs is required. Here, data derived from “omics” studies will help to gain critical insights into the specific metabolic dependencies. Even though this research area is still in its infancy, several studies aimed at addressing altered metabolism in immune cells using omics data and will be detailed below.

## ILC2 omics

### - Transcriptional analysis

Transcriptomics analyses enable the study of gene expression profiles, and it can be applied to distinguish gene expression patterns between healthy and diseased tissues or cells. Here, RNA-sequencing (RNA-seq) offers a powerful tool for immune profiling by identifying specific markers on immune cells in a disease state that can be used as targets for therapeutic approaches. Moreover, this technique aids to identify novel immune cell subsets, including those that are present at low frequencies (Haque et al., 2017). With respect to ILC2s, their identification as well as characterization still remain a major challenge due to their similarities with other immune cells. To overcome this hurdle, one study in 2017 applied a novel multi-class gene expression-based model of single-cell RNA-seq data of natural killer (NK) and ILCs in order to identify ILCs in this mixed population (Suffiotti et al., 2017). For their analysis, the group used mouse gene expression data obtained from Robinette et al. (2015). In their study, they identified several subclusters of NK cells, ILC1s and ILC3s, whereas ILC2s were defined as the most distinguishable class (CD127^+^, Sca-1^+^, and ST2^+^) isolated from the lamina propria in the small intestine of 6-week-old C57BL/6 male mice (Robinette et al., 2015). Due to that, in the multi-class gene expression-based model from 2017, ILC2s achieved the best predictions and can be studied at the gene expression level even though ILCs are present at a lower frequency as compared to NK cells (Suffiotti et al., 2017). Moreover, they were able to find ILC2s in a pool of bulk single-cell transcriptomics data by applying this new classifier. Even more striking, all of the predicted ILC2s represent actual ILC2s, showing that their model offers a reliable tool to accurately identify this specific cell-type. Thus, this model allows for a computation framework to study ILC2s in published datasets without the use of specific antibodies (Suffiotti et al., 2017).

In a study conducted in 2022, the technologies of RNA-seq and Assay for Transposase-Accessible Chromatin using sequencing (ATAC-seq) have been combined to obtain information about the three-dimensional (3D) genome organization required for the development and function of murine ILC2s in steady state and during allergic aiway inflammation (Michieletto et al., 2023). With this approach, the authors could show how local spatial configuration of the genome significantly affects ILC2 biology. Specifically, they found that the development of ILC2s and the progression of allergic airway inflammation are governed by a distinctive 3D configuration which contained the ILC-lineage-defining factor *Id2* (Michieletto et al., 2023). Here, multiple interactions between the *Id2* promoter and distal regulatory elements bound by the transcription factors GATA3 and RORα reveal an important mechanism for ILC2 development and homeostasis. Moreover, they found binding sites for transcription factors that are required for ILC2 function and development, such as ETS proto-oncogene 1, transcription factor (ETS1) and RUNX family transcription factor 1 (RUNX1). In conclusion, both studies provide an in-depth analysis to improve ILC2 identification and functions (Suffiotti et al., 2017; Michieletto et al., 2023).

Since ILC2s show a vast adaptation to environmental cues depending on the tissue, one group aimed at characterizing the impact of these stimuli (e.g., cytokine production, lipid mediators, nutrients or hormones) on ILC2 immunity in different human tissues of healthy donors (blood, tonsil, lung and colon) using single-cell RNA-seq (Mazzurana et al., 2021). The annotation of their identified ILC2 clusters was based on a previous study, which intended to analyze the heterogeneity of ILCs in human tonsils (ILC2: Lin^−^CD127^+^CRTH2^+^ST2^+^CD25^+^KLRG1^+^GATA3^+^) (Bjorklund et al., 2016). In brief, they found tissue-specific signatures of ILCs with respect to migration, activation as well as modified metabolism. Interestingly, they could also reveal differentiation pathways from circulating and tissue-resident naive ILCs to fully differentiated ILC subsets (Mazzurana et al., 2021). Moreover, they were able to identify a novel subcluster of lung-resident ILC2s (CRTH2^-^ ILC2). Chemoattractant receptor-homologous molecule expressed on TH2 cells (CRTH2) has been shown to be required for ILC2 activation and cytokine production (Xue et al., 2014). However, in their study they observed a new acquired phenotype by ILC2s upon exposure to tissue-derived alarmins, suggesting that activated ILC2s in the lung do not depend on CRTH2 expression for their functionality (Mazzurana et al., 2021). In turn, pulmonary ILC2s also express CCR8, a receptor recently found to control the homeostasis of Tregs via non-canonical effects involving CCL17 and CCL3 (Döring et al., 2024; Puttur et al., 2019) Overall, this work enhances the potential of single-cell RNA-seq to characterize ILC2s in a tissue-specific manner. Importantly, their single-cell RNA-seq dataset together with two others was used to perform an integrative interference analysis of ILCs in order to assess their transcriptional imprinting across six different human tissues including healthy and diseased tissues (Song et al., 2023). They could identify four ILC2 subsets with different tissue distributions. Two subsets were identified as c-Kit^+^ ILC2s and only occurred in hepatocellular carcinoma (HCC), resulting from plasticity in the tumor environment. In line with this, another study reported an increased frequency of the c-Kit^+^ ILC2 population in HCC-derived supernatant with high IL-6 and TGFβ (Heinrich et al., 2022). One Killer cell Lectin-like receptor G1^+^ (KLRG1^+^) ILC2 subset was predominantly found in blood, and ILC2s expressing the tissue-residency marker CD69^+^ showed the highest expression in tonsil. The latter subset also displayed high levels of regulators for an anti-inflammatory immune response (i.e., Dual Specificity Phosphatase 1 (*DUSP1)* and Early growth response protein 1 (*ERG1*)). Intriguingly, circulating ILC2s expressing KLRG1 from patients with allergic rhinitis did not produce the anti-inflammatory cytokine IL-10, which could be restored upon allergen immunotherapy (Song et al., 2023; Golebski et al., 2021). Such analyses are still lacking for metabolic and CVDs but would help to obtain a better understanding of the similarities and differences of ILC subsets in the different tissues and give important insights into their phenotypical characteristics and how they change in a diseased state.

In this regard, Jiang *et al.* conducted a study to reveal the role of infiltrating ILC2s in ischemic myocardium (Jiang et al., 2023). Specifically, they applied single-cell RNA-seq on heart tissues of 8-week-old male C57BL/6J mice that were subjected to myocardial infarction (MI) and myocardial ischemia-reperfusion injury (MIRI) (Jiang et al., 2023). Importantly, they could identify five ILC2 subsets - ILC1, ILC2a, ILC2b, ILCdc and ILCt, from which the last two were unique for heart tissue. ILC2a and ILC2b showed high expression of typical ILC2 markers (i.e., *Klrg1*, *Gata3*, *Rora*, *Il5*, *Amphiregulin (Areg*)). However, ILC2b expressed in addition to that *Stathmin 1 (Stmn1)*, *Baculoviral IAP Repeat Containing 5 (Birc5)* and *PCNA Clamp Associated Factor (Pclaf)*, which represent cell-cycle-associated genes. Moreover, they could find genes related to proliferation, overall suggesting that the ILC2b cluster is required for the proliferation 3 days after MI Interestingly, the ILCdc cluster showed expression of ILC-reg-related genes, such as Id3 (Jiang et al., 2023; Thomas and Peebles, 2022). Additionally, the ILCt cluster expressed signature genes of T cells, suggesting that this subset might act in parallel with T cells. Moreover, they published a ligand–receptor–transcription factor–target gene regulatory network to reveal crosstalk between the different ILC clusters (Jiang et al., 2023). By applying this network analysis, they revealed communications among the clusters and found that the cluster of ILCdc received signals from all other clusters, whereas ILCdc-derived messages only reached to itself and to ILC2b. This communication might be important to dissect the specific roles for each ILC subcluster in myocardial ischemia diseases. Furthermore, the identification of tissue- and disease-specific ILC2 subsets can be used as potential targets for the treatment of myocardial ischemia diseases by allowing a more specific targeting approach for the relevant ILC subset. Overall, such studies help to gain critical new insights into the functional adaptation of ILC2 at different tissue sites and in disease and reveal novel strategies for treatment approaches.

In order to provide in-depth single-cell analysis, mass cytometry, also known as CyTOF (Cytometry by Time-Of-Flight) offers a powerful tool to decipher complex biological systems and to study immune cell diversity, among others. This technology combines flow cytometry principles with mass spectrometry, thereby enabling measurements of multiple parameters simultaneously (Zhang et al., 2020). In this regard, Zernecke *et al.* published a meta-analysis of leukocyte diversity based on published data using either single-cell RNA-seq or CyTOF technologies from atherosclerotic aortas of mice (Zernecke et al., 2020). The data is derived from published studies using different mouse models to obtain atherosclerotic aortas: two genetic knockout models - *Ldlr* ^−/−^ (low-density lipoprotein receptor knockout) (Cochain et al., 2018; Kim et al., 2018) and *Apoe* ^*−/−*^ (Winkels et al., 2018), or adeno-associated virus PCSK9 (protein convertase subtilisin/kexin type 9) induced lipoprotein changes (Lin et al., 2019). Importantly, in their analysis they could find an ILC2 cluster displaying signature genes such as *Areg* (encoding amphiregulin), *Il1rl1* (encoding the IL-33 receptor ST2), RORA and GATA3 in datasets of Cochain et al. (2018) and Winkels et al. (2018). More importantly, the presence of this ILC2 cluster could be related with data from previous work revealing their atheroprotective functions (Newland et al., 2017). In this study, they found that ILC2-deficient mice displayed an accelerated progression to atherosclerosis, which was partly due to ILC2-derived IL-5 and IL-13. Moreover, they found profound phenotypical changes of ILC2s in tissues of mice fed a high-fat diet. Specifically, they observed that ILC2s derived from mesenteric and para-aortic lymph nodes of old *Apoe* ^*−/−*^ mice had low ST2 expression and thus, resembled more the inflammatory phenotype of ILC2s as described by Newland et al. (2017), Huang et al. (2015).

Combined, it is clear that transcriptomic approaches can be a valuable tool to further elucidate the complex regulatory mechanisms and functionality of ILC2s, although more applied studies are needed to gain sufficient insights.

### - Proteomics

In contrast to genomic or transcriptomic studies, proteomics involves the large-scale study of proteins present in a biological sample. Data derived from proteomics analysis for ILC2s can offer additional information regarding the abundance, post-translational modifications, and interactions with other proteins involved in metabolic pathways, energy production, and signaling cascades (Aslam et al., 2017).

In a recent study, quantitative mass spectrometry-based proteomics was applied to analyze proteins expressed by ILC2s and ILC3s obtained from healthy human skin and blood of donors (Teunissen et al., 2024). Briefly, ILCs were identified as CD45^+^Lin^−^CD127^+^CD161^+^ (Vivier et al., 2018) and ILC2s were further distinguished from ILC3 by the expression of CRTH2 (Teunissen et al., 2024). Their proteomic analysis identified cluster of differentiation marker profiles of the ILC subset and further allowed to study their distribution and abundance of known proteins. Specifically, they could find proteins that were differentially expressed between these two subsets in the skin. For instance, they found prostaglandin D synthase (HPGDS) and CRTH2 to be exclusively expressed by ILC2s. Furthermore, they could find novel subset-specific protein signatures. Importantly, the data set confirmed the expression of ILC2-specific proteins, which are also expressed at the mRNA level, such as GATA3 and CD161 (Vivier et al., 2018). Intriguingly, ST2 and TSLPR cytokine receptors could not be identified in ILC2s in their proteomic study (Teunissen et al., 2024). Accordingly, this finding is in accordance with another study which could also not identify these receptors on human blood-derived ILC2s of healthy individuals (Bal et al., 2016). It is important to note that, most phenotypical characteristics of ILC2s derive from studies using inflamed tonsils, which is therefore most likely the cause of the observed discrepancies (Vivier et al., 2018). These discrepancies in ILC2 phenotypes enhance the importance to study tissue- and disease-specific characterizations of ILC2s.

Therefore, combining transcriptomics and proteomics data would provide a more comprehensive understanding of biological systems by bridging the gap between gene expression and protein activity. While transcriptomic studies reveal mRNA levels for potential protein expression, data derived from proteomics could confirm the actual abundance of a specific protein. Additionally, the combination of these approaches would also enable the identification of novel markers for a more accurate characterization of ILC2s.

### - Metabolomics

Similar to proteomics, metabolomics focuses on large-scale analysis of proteins. However, more specifically, metabolomics is the comprehensive study that gives insights into small molecules, or metabolites that are present in a biological sample. With respect to ILC2s, these data can reveal important insights into the metabolic pathways that are required for their activation and function, as well as the abundance of certain metabolites or any metabolic changes.

For instance, Surace *et al.* gave important insights into the dichotomous metabolic networks of proliferation and effector function of human blood ILC2s obtained from healthy donors (Surace et al., 2021). Specifically, the authors could reveal that circulating ‘naive’ ILC2s exhibit an unanticipated metabolic profile, showing higher levels of OXPHOS as compared to NK cells. Additionally, these circulating ILC2s did not express ST2. Intriguingly, it has been shown that IL-33 induced ILC2 activation and proliferation, led to an increase in glycolysis to produce IL-13 while maintaining OXPHOS for their cellular fitness and proliferative capacity (Surace et al., 2021). Importantly, these different metabolic pathways offer various angles to therapeutically manipulate ILC2s in a diseased state. Accordingly, targeting immunometabolism has emerged as promising approach to control both inflammatory and anti-inflammatory immune responses. Here, specific metabolic events can be used to dampen inflammation (reviewed in (Pålsson-McDermott and O’Neill, 2020)). For instance, metformin is used for the treatment of T2D due to its action in reducing hepatic gluconeogenesis and it has been shown to affect macrophages, T cells and B cells (Kim et al., 2008; Diaz et al., 2017; Kelly et al., 2015; Zarrouk et al., 2014). Modulating key metabolic pathways by inhibiting or activating glycolysis or OXPHOS could give important insights into ILC2 activity. This can be achieved by applying drugs that specifically target metabolic pathways or enzymes involved in ILC2 metabolism. For instance, 2-deoxyglucose (2-DG) is a nonmetabolizable glucose analog and can be used as inhibitor for glycolysis (Nakada and Wick, 1956). Notably, 2-DG is intensively studied as potential anti-cancer agent (Raez et al., 2013; Stein et al., 2010; Xi et al., 2014; Zhang et al., 2014) and it has been approved as emergency drug in 2021 for the treatment of COVID-19 (Aiestaran-Zelaia et al., 2022). However, studies looking at the requirements for modulating ILC2 metabolism remain scarce and thus need to be further explored.

Another study used an unbiased metabolomics analysis of feces derived from mice infected with *N. brasiliensis* (*Nippostrongylus brasiliensis*) to reveal how environmental and metabolic stimuli shape ILC2 immune responses upon helminth infection (Hodge et al., 2023). Interestingly, they found an increase of essential amino acids upon infection. To further scrutinize the importance of the availability of these amino acids for ILC2 function, they fed the mice a low protein diet after infection. Indeed, ILC2 numbers were reduced in those mice fed a low protein diet by day 7 after the infection with *N. brasiliensis*, thereby suggesting that amino acid availability impacts ILC2 immune responses (Hodge et al., 2023).

## Summary and conclusion

Overall, the integration of multiple omics approaches, including transcriptomics, proteomics, and metabolomics can provide a comprehensive understanding of the metabolic profile and functional characteristics of ILC2s in health and chronic vascular inflammation. Table 1 provides an overview of the different omics fields that can be applied to reveal the function and characteristics of ILC2s with respect to metabolic and CVDs. It is important to note that the specific experimental conditions, sample preparation methods, and data analysis techniques should be taken into consideration when interpreting omics data for metabolic profiling of immune cells like ILC2s.

**TABLE 1:** Overview of ILC2 focused omics approaches.

Omics	Studies in ILC2s	Relevance to inflammation and metabolic diseases	References
Genomics	Identification of genetic variants and susceptibility loci associated with ILC2 function and regulation	Genetic predisposition to cardiovascular inflammation and atherosclerosis linked to ILC2-related genes	Liu et al. (2022), Xu et al. (2023)
Epigenomics	DNA methylation and histone modification patterns in ILC2s during cardiovascular stress	Epigenetic regulation of ILC differentiation and function affecting cardiovascular disease progression and response to therapy	Michieletto et al. (2023)
Metabolomics	Metabolic profiling of ILCs revealing unique metabolic pathways and metabolites in a disease- and tissue-specific context	Understanding how metabolic states of ILC2s influence their function in cardiovascular pathology, including oxidative stress and impaired lipid metabolism	Surace et al. (2021)
Transcriptomics	Differential expression of key genes in ILC2s under inflammatory conditions	Insights into the phenotypical characteristics of ILC2s in promoting or mitigating atherosclerosis	Jiang et al. (2023), Zernecke et al. (2020)
Proteomics	Characterization of protein expression profiles and signaling pathways in ILC2s	Identification of protein markers and signaling molecules involved in ILC2-mediated vascular inflammation	Teunissen et al. (2024)
Multi-Omics Integration	Combined analysis of genomic, transcriptomic, proteomic, and metabolomic data in ILC2s	Holistic understanding of ILC contribution to CVDs, leading to the identification of novel therapeutic targets and biomarkers	as applied in (Song et al., 2023)

Nevertheless, integrating omics data from different immune cell types provides critical insights into the complex interactions between immune cells and vascular tissues in health and disease. Furthermore, dissecting this interplay together with the cytokine networks will be crucial for a better understanding of inflammatory processes driving vascular pathologies. Even further, network analysis based on omics data can reveal molecular players and signaling pathways linking ILC2s to CVDs. In this regard, Fabian Theis’ group just recently published a novel method to model intercellular communication in tissues using spatial graphs of cells, the so-called node-centric expression models (NCEM) (Fischer et al., 2023). These graph-based neural networks aim at revealing the interplay between tissue niches and gene expression without losing spatial information.

Such advances in omics data have revolutionized the field of life sciences, allowing for a deeper understanding of the complex interactions within biological systems. Moreover, the integration of multi-omics data will aid in developing new diagnostics, therapeutics, and personalized medicine approaches.
